# Trypanosomes and complement: more than one way to die?

**DOI:** 10.1016/j.pt.2023.09.001

**Published:** 2023-12

**Authors:** Alexander D. Cook, Mark Carrington, Matthew K. Higgins

**Affiliations:** 1 Department of Biochemistry, University of Oxford, South Parks Road, Oxford OX1 3QU, UK; 2 Kavli Institute for Nanoscience Discovery, Dorothy Crowfoot Hodgkin Building, University of Oxford, South Parks Rd, Oxford, OX1 3QU; 3 Department of Biochemistry, University of Cambridge, Tennis Court Rd, Cambridge CB2 1QW, UK

**Keywords:** African trypanosome, complement system, ISG65, complement factor C3

## Abstract

African trypanosomes show a remarkable ability to survive as extracellular parasites in the blood and tissue spaces of an infected mammal. Throughout the infection they are exposed to the molecules and cells of the immune system, including complement. In this opinion piece, we review decades-worth of evidence about how complement affects African trypanosomes. We highlight the discovery of a trypanosome receptor for complement C3 and we critically assess three recent studies which attempt to provide a structural and mechanistic view of how this receptor helps trypanosomes to survive in the presence of complement.

## Highlights

African trypanosomes replicate in the blood of mammals, where they must resist destruction by the complement system.
Trypanosomes have evolved multiple mechanisms to reduce complement-mediated killing, including a dense cell surface coat, rapid cell surface clearance and receptors (including ISG65) for complement components.
Structural and functional studies have revealed how ISG65 binds to complement C3 and its derivatives.
Three recent studies propose different mechanisms by which ISG65 might reduce the effects of complement, based on *in vitro* assays.
Uncovering the mechanism of action of ISG65 will require studies of its effect on trypanosome resistance to complement in an infection model.

## Are trypanosomes killed by complement?

**African trypanosomes** (see Glossary) are parasites which have evolved to flourish in the blood of their mammalian hosts. Indeed, in some cases, they maintain chronic infection for decades, despite simultaneous attack from the varied branches of the mammalian immune system. How do they survive?

One of the primary, and most ancient, mechanisms of mammalian immunity is the **complement system** (Box 1) [1]. This set of protein factors centres around the deposition of **complement factor C3b** on a pathogen surface, which occurs after reaction of a thioester in C3b with a cell-surface component, usually with a hydroxyl group [2]. Deposition can occur through three interlinked processes: (i) spontaneous deposition (the ‘alternative’ pathway), (ii) deposition mediated by antibodies (the ‘classical’ pathway), or (iii) carbohydrate recognition (the ‘lectin’ pathway) [3]. Deposited C3b can then initiate different molecular and cellular mechanisms, through which the pathogen is destroyed, including formation of pores in the pathogen membrane or stimulation of immune cell activity.

Box 1 **The complement system**

The complement system is a protein cascade that triggers a varied antipathogen response at foreign surfaces (Figure I). Inactive complement proteins circulate in the blood and are activated by a sequence of tightly regulated proteolytic events. Activation of complement is centred around the conversion of complement factor C3 to C3b. This exposes reactive thioester forming residues, which can form covalent thioester bonds with a neighbouring pathogen surface. Amplification of the amount of pathogen-bound C3b occurs via C3 convertases. These are short-lived enzymes that are subject to host regulation to ensure that C3b deposition is amplified only on pathogen surfaces. Generation of C3b releases inflammatory molecules (anaphylatoxins), coats the pathogen with immune-visible molecules (detected via complement receptors), and catalyses formation of the pore-forming membrane attack complex (MAC).

The complement system can be specifically activated by the classical pathway (triggered when complement C1 recognises antibody-coated pathogens) and the lectin pathway [triggered when the lectin, mannose-binding lectin (MBL), recognises pathogen-specific sugars]. Both pathways generate the pathogen-bound enzyme C4bC2b. This is a C3 convertase that cleaves C3 to C3b, which then forms a thioester bond with the pathogen surface and it can be negatively regulated by C4 binding protein (C4BP). C3b accumulation triggers the alternative pathway, where factor B (FB) binds to C3b and is activated by factor D (FD), forming the second C3 convertase C3bBb. An amplification loop then proceeds, leading to more C3b deposition, which is positively regulated by properdin (P) and negatively regulated by factor H (FH). The alternative pathway may also be triggered nonspecifically by a conformational change in C3 to a C3b-like molecule, without any cleavage event. This may be spontaneous or stimulated by contact with surfaces.

Complement gives rise to diverse immune outcomes. Surface-bound C3 convertases can bind additional surface-bound C3b molecules and generate a new enzyme with much higher affinity for C5. These C5 convertases [C3bBb(C3b)_n_ and C4bC2b(C3b)_n_] can cleave C5 to C5b, triggering assembly of the MAC, resulting in cell lysis. Cleavage of C3 to C3b, and C5 to C5b, also releases C3a and C5a. These are anaphylatoxins which bind to specific receptors (C3aR and C5aR) on lymphoid cells and act as powerful chemoattractants. Deposited C3 truncations can also stimulate immune cell activity. Recognition of C3b by complement receptor CR1 on immune cells promotes immune adherence and phagocytosis. C3b can be further proteolysed by the action of FH and factor I (FI) to iC3b, C3dg, and C3d. These fragments are recognised by complement receptors CR3 and CR4, which are found on various leukocytes, and by complement receptor CR2, which is a part of a B cell coreceptor which plays a crucial role in B cell memory development.

**Figure I:**
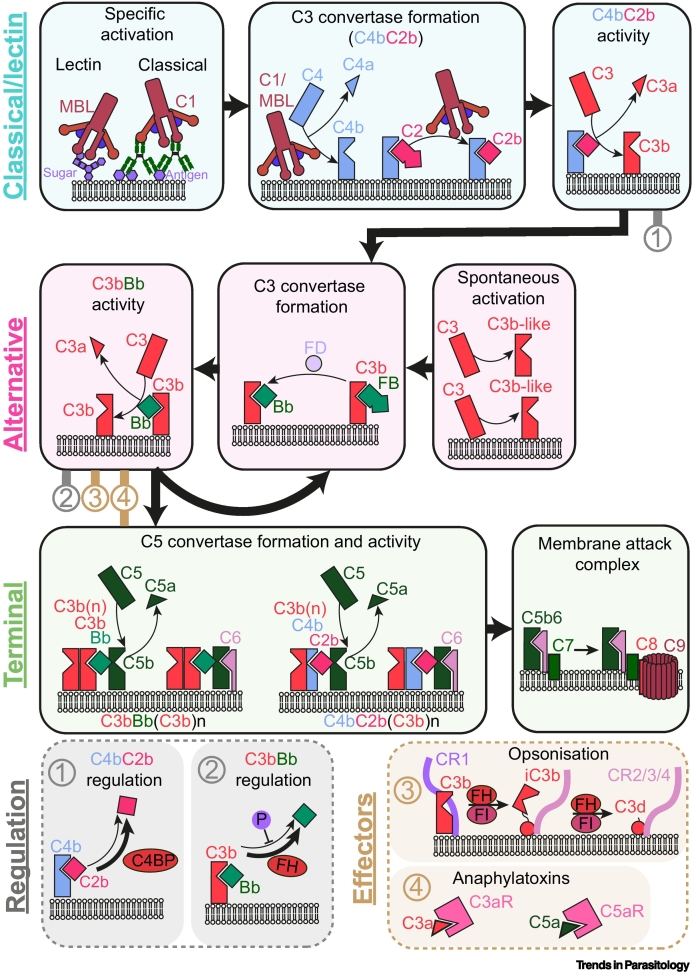
The complement system. Abbreviations: C4BP, C4 binding protein; FH, factor H; FI, factor I; MBL, mannose-binding lectin.

Alt-text: Box 1

Trypanosomes, as extracellular pathogens of the blood, are constantly exposed to components of the complement system, with which they have long coevolved. This raises the question of how they maintain an infection in the presence of complement. Does complement reduce trypanosome density during infection? Can complement trigger more than one way for a trypanosome to die? And what molecular processes have they evolved to resist destruction?

## The consequences of a remarkable trypanosome cell surface and structure

To understand the interactions that trypanosomes make with complement, we need to understand the remarkable surface coat and cellular structure of the **bloodstream form** trypanosome. To allow effective **antigenic variation**, each cell is covered with a dense layer of many copies of a single **variant surface glycoprotein (VSG)** within which other cell-surface proteins operate [4]. This VSG layer protects the underlying plasma membrane, and switching to an antigenically distinct VSG allows a trypanosome population to avoid immune clearance [5].

The trypanosome cell structure is also unusual. Trypanosomes are elongated cells with a single flagellum emerging from an invagination of the plasma membrane towards their posterior end. This flagellum runs along the entire cell and extends beyond the anterior end of the cell body. The forward swimming of the cell drives hydrodynamic flow across the cell surface, sweeping components that protrude above the VSG layer towards the flagellar pocket, which is the sole site of endocytosis. Antibodies which attach to the cell surface are therefore cleared rapidly [6].

These features have consequences for the action of the complement system. Does the VSG layer reduce C3b conjugation to any cellular component other than VSGs? Does hydrodynamic flow clear C3b-conjugated VSGs from the cell surface? Can the **membrane attack complex (MAC)** penetrate the dense VSG layer to form pores? Is complement able to help protect against trypanosome infection?

## Are trypanosomes killed by the classical pathway of complement?

Mammalian bloodstream forms of the best studied African trypanosome, *Trypanosoma brucei,* replicate *in vitro* in fresh serum from an uninfected mammal. This serum contains active complement components but no antibodies against the trypanosome surface. By contrast, equivalent sera containing antibodies against the expressed VSG causes trypanosome killing [7]. Indeed, this technique of *in vitro* killing with specific VSG antibodies and serum was widely used in experiments which aimed to select cells expressing novel VSGs [8,9] and causes direct trypanosome lysis as well as recruitment of macrophages [7]. As this killing requires both serum and antibodies, it was understood to be occurring due to the action of the **classical pathway of complement**. Indeed, incubation of *Trypanosoma congolense* with bovine serum led to the formation of VSG-C3b conjugates detected only when antibodies against VSG were present, suggesting that C3b recruitment was mediated through the classical pathway [10].

Studies of trypanosomes in mammalian infection models have also provided evidence for the action of the classical pathway of complement. When a mouse is infected with trypanosomes which express a single VSG, a first wave of infection occurs in which trypanosome cell density increases and decreases again. This decrease in trypanosome cell density is antibody dependent, as it does not occur in mice lacking B cells (μMT^–/–^) [11]. Antigenic variation then results in a trypanosome population which is no longer recognised by the antibodies induced by the first wave, resulting in a second wave of trypanosome growth. Recently, we also showed that the decrease in trypanosome growth in the first wave of infection does not occur in mice lacking **C3** [12]. Therefore, the control of the first wave of trypanosome infection, occurring before antigenic variation, is dependent on both antibodies and complement C3. Together, these experiments with knockout mice indicate that this decrease in trypanosome cell density is due to the classical pathway of complement [12].

## Are trypanosomes killed by the alternative pathway of complement?

As soon as a trypanosome infects a mammal it is exposed to stochastic activation of the **alternative pathway** of complement. Does this play a role in the control of infection?

Several studies have investigated binding of complement components to trypanosomes in the absence of antibodies. While one found C3b-VSG conjugates to occur only in the presence of VSG-targeting antibodies [10], another showed labelling of trypanosomes with iodinated C3b-binding antibodies in the absence of VSG-targeting antibodies, suggesting deposition through the alternative pathway [13]. However, this study did not show whether the C3b was on the trypanosome surface, able to trigger downstream processes associated with cell killing, or whether it is rapidly endocytosed and inactivated. A recent study confirmed association of C3b and factor B with trypanosomes, showing that, in this case, both were located primarily within the endomembrane system [14]. Therefore, the alternative pathway can cause accumulation of C3b in trypanosomes. But is C3b conjugated to the cell surface, and does it contribute to trypanosome killing?

Studies of trypanosome growth, either *in vitro* or *in vivo*, have not shown cell killing through the alternative pathway of complement. Many laboratories culture trypanosomes in serum in which complement has not been inactivated through heating. In the absence of cell-surface interacting antibodies, these cells proliferate, suggesting that alternative-pathway-mediated deposition of C3b is not sufficient to allow killing [15]. In a mouse model, we also see no effect from the alternative pathway. During the initial phase of trypanosome growth, which occurs in the 3–4 days post-infection, there are no antibodies against the trypanosome surface, and the classical pathway will not take place. However, the alternative pathway will be active. During this period, trypanosomes proliferate at the same rate in wild type and C3-deficient mice [12]. This suggests that, if C3b is deposited during this period through the alternative pathway, then it does not affect trypanosome proliferation.

## Does the trypanosome surface protect from MAC-mediated killing?

One of the major mechanisms of complement-mediated killing involves C3-dependent assembly of the **MAC**, leading to cell lysis [16]. For this to occur, complement components must access the pathogen plasma membrane, allowing MAC-mediated pore formation. Is this blocked by the VSG layer?

Our understanding of whether the MAC can form on a trypanosome has been aided by comparing complement sensitivity of bloodstream-form trypanosomes with that of the **procyclic** developmental form that proliferates in insects and which lacks the VSG coat. While bloodstream-form trypanosomes are resistant to lysis, procyclic forms are rapidly lysed [15]. In addition, trypsinisation of bloodstream forms rendered them susceptible to lysis, presumably due to proteolysis of the VSG coat [17]. A similar picture was revealed by studying a spontaneous mutant *T. congolense* line which lacks the VSG coat and which shows greater susceptibility to lysis [15].

These data suggest that the VSG coat reduces accessibility of the trypanosome membrane to assembly of the bulky MAC, contributing to survival of trypanosomes in response to the alternative pathway. Indeed, in a study in which *T. brucei* is incubated with sera in the absence of surface reactive antibodies, MAC components C5 and C9 were not detected [13]. Whether targeted recruitment of MAC components through the classical pathway can overcome the steric effects of the VSG layer remains to be confirmed. However, the observed lysis of trypanosomes by incubation in serum with surface-reactive antibodies [9] suggests that targeted antibody-mediated recruitment of C3b to the trypanosome surface may overcome the protective effect of VSG and allow trypanosome killing.

## A receptor for C3 and its fragments operates on the bloodstream-form trypanosome surface

**Figure 1:**
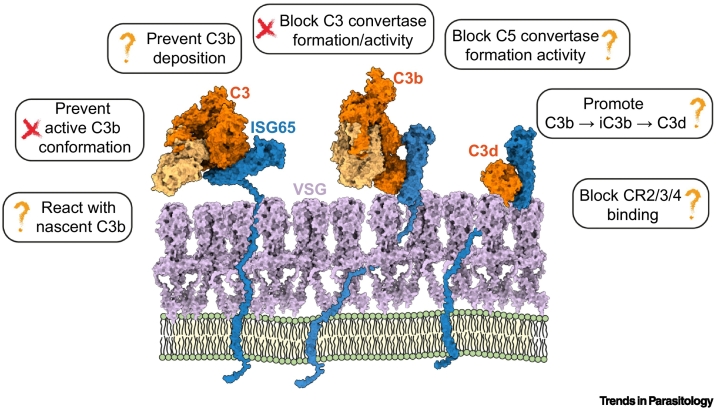
How does ISG65 reduce the effects of complement in African trypanosomes? ISG65 (blue) is attached to the trypanosome plasma membrane (green) via a flexible string. This allows it to reach above the VSG surface (grey), where it can bind to C3 derivatives, including C3, C3b, and C3d. How it reduces the destruction of trypanosomes by the complement system is unknown. Abbreviations: ISG65, invariant surface glycoprotein 65; VSG, variant surface glycoprotein.

In addition to the VSG coat, the trypanosome surface has other adaptations to allow it to resist the effects of complement. Within this coat operate members of a family of three-helical bundle receptors [18., 19., 20.]. These include a gene family coding for multiple copies of **invariant surface glycoprotein 65, ISG65**, which we identified to be receptors for C3 [12] (Figure 1). We also showed that two ISG65 isoforms, representing the extremes in ISG65 diversity across one genome, both bound to C3, indicating that members of the full ISG65 family act as C3 receptors. We also showed that one ISG65 bound to C3 from a range of mammalian species [12]. This discovery was made by our team using genes from *T. brucei brucei* and was later confirmed by a former member of our team using ISG65 from the closely related trypanosome subspecies *T. brucei gambiense* [14]. There are no specific polymorphisms which distinguish ISG65 receptors from *T. b. brucei* and its human-infective subspecies *T. b. gambiense* and *T. brucei rhodesiense* [21], making it likely that complement evasion is conserved across these pathogens.

The next question was whether the presence of ISG65 increases the ability of trypanosomes to resist killing in the context of the complement system. This was again assessed using a mouse infection model [12]. We generated a trypanosome line in which both alleles of the entire tandem array of ISG65 genes were deleted and we compared the growth kinetics of both wild-type trypanosomes and the ISG65^–/–^ line during mouse infection. In both cases, an initial increase in trypanosome numbers was followed by a C3-dependent decrease, with this first wave then followed by a second wave of trypanosomes. Deletion of ISG65 altered the timing of the decrease of the first wave of trypanosomes, with ISG65^–/–^ cells more rapidly controlled [12]. This indicates a role for ISG65s in surviving complement-mediated killing.

ISG65 is the second receptor for a complement component identified in African trypanosomes, following our previous identification of a receptor for factor H [19]. However, the pattern of ISG65 and factor H receptor expression across the trypanosome developmental forms is not the same, with the factor H receptor expressed predominantly in the stumpy developmental form which is transmitted to insects, as well as in insect-resident procyclic forms. Like ISG65^–/–^, a factor H receptor knockout line is cleared more rapidly during the first wave of mouse infection and shows decreased transmission to tsetse flies [19]. We think it likely that there will also be other receptors for complement components which combine with ISG65 and the factor H receptor to facilitate protection as part of a redundant system.

## How does ISG65 bind to and inhibit C3 activity?

The activation and deployment of the complement cascade requires C3 to undergo a range of proteolytic processing events, conformational changes, and interactions (Box 1) [1]. To determine how ISG65 binds to C3, multiple studies measured which forms of C3 bind to ISG65 [12,14,21]. While there are differences in the detailed outcomes of these studies, there is agreement that ISG65 binds to soluble C3 and to its processed C3b form. ISG65 also binds to C3d, which is known as the **thioester domain (TED)** before it is cleaved from C3b, and, after cleavage, aids recruitment of immune cells to a labelled pathogen.

Structural studies have shown how ISG65 binds to C3d, C3b, and C3. First came our crystal structure of ISG65 bound to C3d, showing that ISG65 forms a curved three-helical bundle with C3d bound to the concave side [21]. Our subsequent structure of the ISG65-C3b complex, derived from cryogenic electron microscopy, confirms this interaction, now between ISG65 and the TED domain of C3b [21]. This later structure also identified a second part to the binding surface, with ISG65 loops, which were disordered in the crystal structure, forming an interface with the CUB domain of C3b [21]. A lower resolution structure showed the same overall binding mode [14]. A final structure shows how ISG65 binds to C3, before the large conformational change which occurs on its conversion to C3b [14]. This confirms that ISG65 can bind to the TED of C3, but not the CUB domain.

These structures give important clues about the function of ISG65. First, they show that ISG65 does not prevent C3b from adopting its active conformation, indicating that it will not block the conversion of C3 to C3b [21]. Second, they suggest that ISG65 is likely to be able to bind to C3b that is conjugated to the trypanosome surface [12]. C3b becomes conjugated to the parasite surface through thioester forming residues in its TED [22]. These residues are not contacted by ISG65, such that ISG65s will be able to bind to surface-conjugated C3b (and C3d) [12]. Third, ISG65 is linked to the plasma membrane through a 73-residue-long flexible linker between the C terminus of the helical bundle and the transmembrane helix which links ISG65 to the trypanosome membrane. This led us to propose a model (the ‘banana-on-a-string’ model) in which the flexible linker will be sufficient to allow the curved helical bundle of ISG65 to swing above the VSG coat, patrolling for surface-conjugated C3b and C3d (Figure 1) [21]. Indeed, ISG65 is accessible on the trypanosome surface and is used in rapid diagnostic tests where it reliably predicts African trypanosomiasis patients [23]. These studies combine to show that membrane-associated ISG65 is unlikely to block conjugation of C3b to the trypanosome surface, but instead can bind to membrane-conjugated C3b, perhaps preventing subsequent steps in the complement cascade (Figure 1).

## How does ISG65 modulate C3 function?

The central place of C3b in the complement cascade provides many sites at which a pathogen could intervene to block its function and reduce complement-mediated killing. It could prevent C3b from being deposited on its surface by inhibiting conversion of C3 to C3b. It could stop amplification of C3b deposition by decreasing the half-life of C3 convertases. It could block the formation of the C5 convertases which catalyse assembly of the MAC and pathogen lysis. Or it could prevent complement receptors from binding to deposited C3b, iC3b, C3dg, and C3d, thereby preventing stimulation of the cellular immune system. Whether ISG65 affects one or more of these processes is an active research area and three reports [14,21,24], published this year, provide different answers, using assays conducted *in vitro*.

Does ISG65 prevent C3b deposition? A recent study assessed the deposition of C3b on microtiter plates coated with different substrates, using conditions designed to mimic each of the lectin, classical, and alternative pathways. ISG65 inhibited C3b deposition in the assay which mimics the alternative pathway, but not in those mimicking the classical and lectin pathways [24]. By contrast, two studies show that C3b does accumulate in wild-type trypanosomes incubated with serum in culture [13,14]. One possible explanation for the discrepancy is that this plate-based assay is conducted with an excess of ISG65 in solution. We find that when we incubate ISG65 with C3b in solution, C3b reacts with ISG65 to form a covalent conjugate, which will no longer be able to bind to a coated surface [21]. Perhaps in an alternative pathway mimic assay, which relies on stochastic C3b activation, this mode of inhibition from soluble ISG65 is sufficient, but in an assay in which antibodies or lectin cause more efficient targeting of C3b to a surface, concentrations of ISG65 greater than those tested would be needed to show this effect? Whatever the reason, even if ISG65 does reduce C3b deposition through the alterative pathway, it does not stop association of C3b with trypanosomes in culture [13,14], the formation of C3b-VSG conjugates [10] or C3-mediated trypanosome killing in a mouse model [12].

A different study assessed the serum-mediated lysis of sheep erythrocytes by complement in conditions designed to mimic the alternative and classical pathways, finding that lysis is inhibited by ISG65 only in the alternative pathway assay [14]. This assay measures the endpoint of the complement cascade, studying erythrocyte lysis through formation of the MAC and does not measure whether C3b is deposited on these erythrocytes. It is therefore also possible that formation of covalent conjugates between soluble ISG65 and C3b [21] is responsible for the inhibition observed here, through a route which would not occur if ISG65 were membrane associated.

If ISG65 does not prevent C3b deposition, does it prevent formation of the C3-convertase C3bBb? This would benefit the trypanosome by inhibiting the conversion of C3 to C3b, thereby reducing the deposition of more C3b on the pathogen surface [25]. Two separate studies perform a very similar assay, using purified components to investigate whether the formation of a C3bBb convertase can occur in the presence of ISG65 [21,24]. Both show clear formation and activity of the convertase in solution, through production of factor Bb, and neither show any effect of ISG65 on this activity. While it is possible that this outcome would be different on a membrane, this finding is consistent with structural modelling which indicates that bound ISG65 would not affect convertase formation or function [21], as well as with experiments which show factor B to be associated with serum-incubated trypanosomes [13,14].

One study also claims that ISG65 inhibits C5 convertase formation [14]. This convertase catalyses deposition of C5, leading to formation of the MAC and pathogen lysis. Several pieces of indirect evidence converge here. One is that C5 and C9 are not found to be associated with trypanosome cells in the absence of antibodies [13,14]. Another is that ISG65 can reduce lysis of erythrocytes in an assay which mimics the alternative pathway [14], although it was not shown at which stage inhibition occurred. While C5 convertase inhibition is an attractive hypothesis for ISG65 function, as it would prevent formation of the MAC, direct evidence in support is currently lacking. We therefore cannot be sure whether ISG65 has a role in preventing the documented lack of trypanosome lysis in response to the alternative pathway of complement, or whether this inhibition is due to the VSG coat.

One study also presents *in vitro* evidence that ISG65 might promote the conversion of membrane-associated C3b to iC3b, which is triggered *in vivo* by factor H [26] or by complement receptor 1 [27]. An *in vitro* assay showed that ISG65 increases the rate of factor H-mediated iC3b formation by just 1.5-fold but increases complement receptor 1-mediated iC3b formation by ~20-fold [24]. However, both iC3b and C3d act as binding partners for complement receptors, aiding recruitment of immune cells such as B cells [28]. It is therefore not clear how promotion of iC3b and C3d formation would reduce trypanosome clearance. Instead, structural comparison shows that ISG65 binds to an overlapping site on C3b when compared with complement receptors 2 and 3, and blocking binding of these complement receptors could reduce immune cell recruitment [21].

These studies all show that unveiling mechanisms of complement modulation is complex. The trypanosome surface, with its VSG coat, its rapid surface clearance mechanism, and its membrane anchored ISG65 is very different from the surfaces studied in *in vitro* assays with soluble ISG65. The interpretation of these assays and how they relate to the function of ISG65 on a trypanosome surface is therefore challenging. A full understanding of how ISG65 inhibits C3b function remains to be determined.

## Concluding remarks: more than one way to die and more than one way to survive?

The mammalian complement system brings together many ways in which a pathogen can be destroyed, from its lysis by pore formation to recruitment of the cellular immune system. Coevolution between trypanosomes and the mammalian complement system have therefore resulted in different mechanisms which trypanosomes can deploy to limit their destruction by complement. It seems likely that a densely packed surface coat reduces lysis by limiting formation of the MAC. But the trypanosome has other tricks. The recent identification of receptors for complement components shows another way in which trypanosomes prolong their ability to resist clearance by the classical pathway of complement. While the importance of ISG65 is now clear, the molecular mechanisms by which it interferes with the complement system are only just starting to be uncovered (see Outstanding questions). To unveil this mechanism, the unusual surface coat of the bloodstream-form trypanosomes ensures that it will be necessary to move away from *in vitro* assays to instead study trypanosomes in infection models. Our view is that it is becoming increasingly likely that, while complement provides more than one way for a trypanosome to die, they have also evolved more than one way to survive.

Outstanding questions Do the alternative, classical, or lectin pathways of complement limit trypanosome survival and proliferation *in vivo*?

Which of the three pathways of complement does ISG65 affect *in vivo*?

Does ISG65 reduce C3 deposition onto the trypanosome surface *in vivo*?

Does ISG65 reduce formation of the MAC *in vivo*?

Does ISG65 reduce immune cell recognition of trypanosomes *in vivo*?

Do other complement-evasion mechanisms operate in trypanosomes?

Do these pathways of complement regulation also operate in the primary livestock-infective trypanosomes, such as *T. congolense* and *Trypanosoma vivax*?

Alt-text: Outstanding questions

## Glossary

**African trypanosomes**: a group of single-celled extracellular eukaryotic pathogens.
**Alternative pathway of complement**: a branch of the complement system in which complement factor deposition occurs stochastically and amplifies other pathways.
**Antigenic variation**: a system by which cell surface receptors are exchanged to avoid antibody-mediated detection.
**Bloodstream form**: the developmental form of the African trypanosome which replicates in mammalian blood.
**Classical pathway of complement**: a branch of the complement system in which complement factor deposition is mediated by antibodies.
**Complement C3 and C3b**: different cleavage products of a central component of the complement system.
**Complement system**: a set of protein components which mediate aspects of innate and adaptive immunity.
**Invariant surface glycoprotein 65 (ISG65)**: a trypanosome surface glycoprotein which acts as a receptor for complement C3.
**Membrane attack complex (MAC)**: a pore-forming complex which mediates pathogen lysis in the complement system.
**Procyclic form**: a developmental form of the African trypanosome which replicates in tsetse flies.
**Thioester domain (TED)**: the domain of complement C3 which covalently conjugates to the pathogen surface.
**Variant surface glycoprotein (VSG)**: a trypanosome surface glycoprotein found in high density across the trypanosome surface, central to antigenic variation.
